# Arbuscular mycorrhiza alters the nutritional requirements in *Salvia miltiorrhiza* and low nitrogen enhances the mycorrhizal efficiency

**DOI:** 10.1038/s41598-022-17121-2

**Published:** 2022-11-16

**Authors:** Chunjuan Pu, Guang Yang, Pengying Li, Yang Ge, Thomas Avery Garran, Xiuteng Zhou, Ye Shen, Han Zheng, Meilan Chen, Luqi Huang

**Affiliations:** 1 Nanjing University of Chinese Medicine, Nanjing, 210023 China; 2 State Key Laboratory of Dao-Di Herbs, National Resource Center for Chinese Materia Medica, China Academy of Chinese Medical Sciences, Beijing, 100700 China; 3 China National of Traditional Chinese Medicine Co., Ltd, Beijing, 102600 China

**Keywords:** Arbuscular mycorrhiza, Secondary metabolism

## Abstract

*Salvia miltiorrhiza* Bunge (*danshen* in Chinese) is one of the most important medicinal cash crops in China. Previously, we showed that arbuscular mycorrhizal fungi (AMF) can promote *S. miltiorrhiza* growth and the accumulation of bioactive compounds. Fertilization may affect mycorrhizal efficiency, and appropriate doses of phosphate (P) and nitrogen (N) fertilizers are key factors for obtaining mycorrhizal benefits. However, the optimal fertilization amount for mycorrhizal *S. miltiorrhiza* remains unclear. In this study, we studied the effects of AMF on the growth and bioactive compounds of *S. miltiorrhiza* under different doses (low, medium, and high) of P and N fertilizer. The results showed that the mycorrhizal growth response (MGR) and mycorrhizal response of bioactive compounds (MBC) decreased gradually with increasing P addition. Application of a low (N25) dose of N fertilizer significantly increased the MGR of mycorrhizal *S. miltiorrhiza*, and a medium (N50) dose of N fertilizer significantly increased the MBC of phenolic acids, but decreased the MBC of tanshinones. Our results also showed that the existence of arbuscular mycorrhiza changes nutrient requirement pattern of *S. miltiorrhiza*. P is the limiting nutrient of non-mycorrhizal plants whereas N is the limiting nutrient of mycorrhizal plants.

## Introduction

*Salvia miltiorrhiza* Bunge (*danshen* in Chinese) is one of the most important medicinal cash crops in China and is used for treating cerebrovascular and cardiovascular diseases^1^, hypertension^2^, ischemic stroke^3^, breast cancer^4^, and hepatitis^5^. *S. miltiorrhiza* is mostly grown under continuous cropping cultivation, which has resulted in serious soil-borne diseases. The high incidence of soil-borne diseases has resulted in the decline of *S. miltiorrhiza* yield and quality, leading to substantial agricultural and economic losses^6^.

Arbuscular mycorrhizae are one of the most important symbiotic relationships in terrestrial ecosystems. Arbuscular mycorrhizal fungi (AMF) form a symbiosis with the roots of more than 90% of terrestrial plants, presenting great nutritional and ecological importance^7^. Many studies have reported that AMFs promote plant growth, nutrition uptake (especially phosphate), and increase resistance to biotic and abiotic stresses^8–12^. AMF can also increase the contents of bioactive compounds such as terpenoids, alkaloids, and phenolics in many economically and industrially significant crops and herbs^13–15^. Our previous study also showed that inoculation with *Glomus mosseae* improves growth and salvianolic acid B accumulation in continuously cropped *S. miltiorrhiza*^15^. However, mycorrhizal efficiency varies with soil fertilizers. The content of phosphate (P) and nitrogen (N) in soil significantly affects the interaction between AMF and plants. In fact, some negative outcomes have also been recorded. For example, P and N fertilization may alter the dynamics of relationships between AMF and plant roots, to reduce the mutualism and even parasitism of AMF^16,17^. Many studies have shown that varying levels of P and N can have different impacts on the benefits that a plant might receive by altering the relationship dynamics of AMF and plants^18,19^, and that mycorrhizal benefits mainly occur under lower P levels^20^.

Rational use of fertilizers is necessary to maximize mycorrhizal benefits. P and N fertilization may cause various mycorrhizal effects on crops, including positive and negative^21^. However, the optimal fertilization amount for mycorrhizal *S. miltiorrhiza* remains unknown. In order to investigate the optimal amount of N or P fertilizer, mycorrhizal *S. miltiorrhiza* and non-mycorrhizal *S. miltiorrhiza* were applied with different levels (low, medium, and high) of N or P fertilizer, respectively. We determined the effects of different levels of P and N fertilizer treatment on the percentage of root length colonized (RLC), biomass of shoots and roots of *S. miltirrhiza*, and the contents of phenolic acids (rosmarinic acid, salvianolic acid B), and tanshinones (dihydrotanshinone, cryptotanshinone, tanshinone I, tanshinone IIA) in *S. miltiorrhiza* root. We also evaluated mycorrhizal efficiency based on the mycorrhizal growth response (MGR) and mycorrhizal response of bioactive compounds (MBC) in the root. The appropriate amount of N and P fertilizer could be revealed in the process of mycorrhizal *S. miltiorrhiza* cultivation, so as to provide reference for the artificial cultivation of *S. miltiorrhiza*.

## Results

### Mycorrhizal colonization was decreased by high levels of P fertilizer but increased by N fertilizer

**Figure 1:**
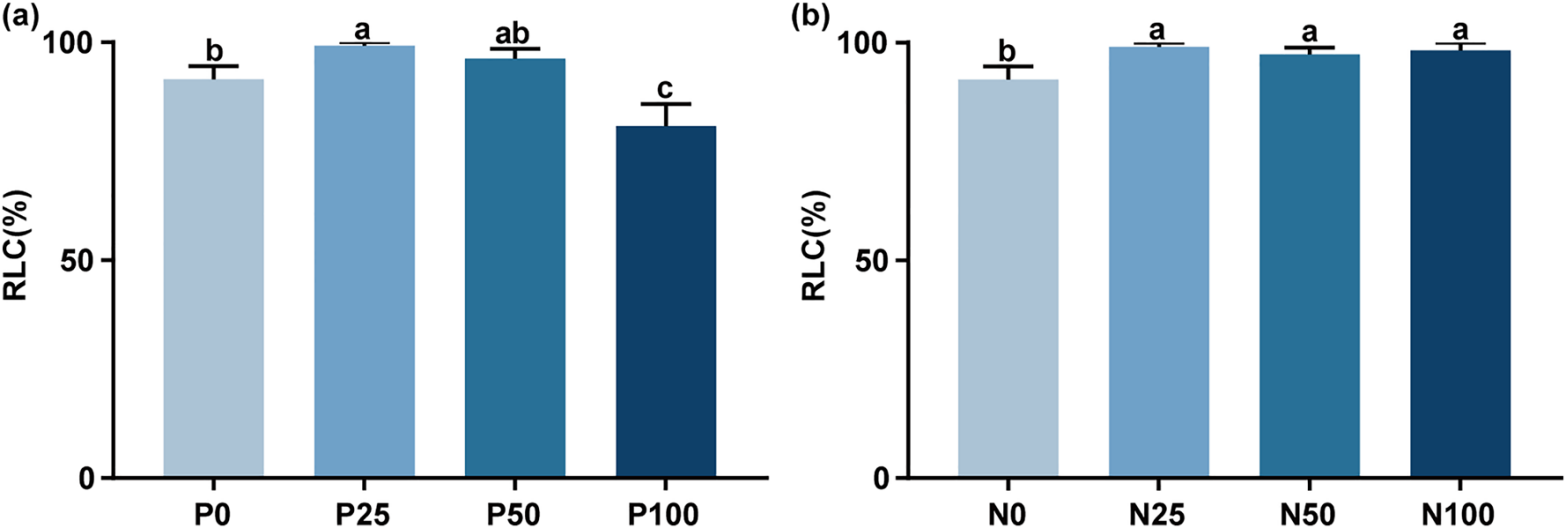
Mycorrhizal colonization of *S. miltiorrhiza* inoculated with *G. versiforme* affected by the addition of P (**a**) and N (**b**) fertilizer. (**a**) The P0, P25, P50, and P100 treatments represent the four levels of P fertilizer application (0, 25, 50, and 100 mg P fertilizer added per kilogram of soil, respectively). (**b**) The N0, N25, N50, and N100 treatments represent the four levels of N fertilizer application (0, 25, 50, and 100 mg N fertilizer added per kilogram of soil, respectively). Percentage of root length colonized (RLC, %) is expressed as the percentage of root length occupied by mycorrhizal fungal structures such as hyphae, arbuscules, and/or vesicles. Means ± standard errors are shown (n = 6). Different lowercase letters indicate significant differences between different treatments according to one-way ANOVA followed by followed by Tukey’s test for multiple comparisons (*P* < 0.05). The roots of control non-mycorrhizal plants remained free of mycorrhizal fungal structures (data not shown).

The percentage of the root length colonized (RLC, %) was used as an index to evaluate root colonization by AMF. The roots of all mycorrhizal plants were highly colonized with AMF, whereas those of non-mycorrhizal plants remained free of AMF colonization. According to the statistical results presented in Fig. 1, AMF colonization was affected by P and N fertilization.

The dosage of P fertilizer significantly affected mycorrhizal colonization. The RLC of the P25 treatment was the highest among all P fertilizer treatments, up to 99.19%, and was significantly higher than that of the P0 treatment (Fig. 1a). In contrast, the RLC of the P100 treatment decreased to 80.79%, which was significantly different from other treatments. This indicates that excessive phosphorus fertilizer is not conducive to establish symbiosis between AMF and plants.

In contrast, N fertilizer application increased mycorrhizal colonization. Applying N fertilizer increased the RLC from 91.52% to 99.04% (Fig. 1b). The N25, N50 and N100 treatments all significantly increased the RLC compared with that of the N0 treatment, and there was no significant difference among the three treatments.

### Application of P reduced the mycorrhizal benefit to plant growth

**Figure 2:**
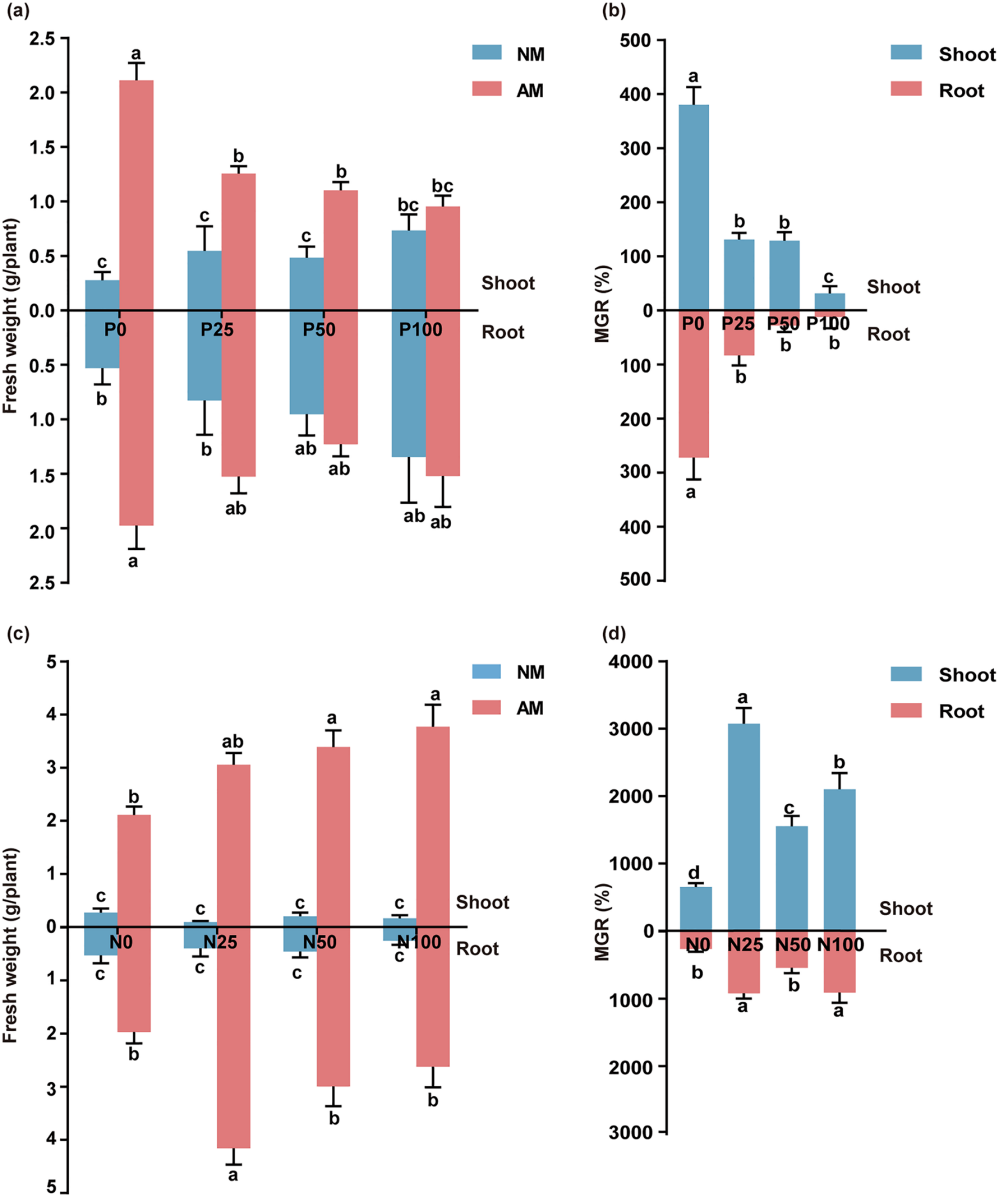
Mycorrhizal efficiency on plant growth under different levels of P (0, 25, 50, and 100 mg P fertilizer added per kilogram of soil; P0, P25, P50, P100) and N (0, 25, 50, and 100 mg N fertilizer added per kilogram of soil; N0, N25, N50, N100) fertilizer. (**a,c**) The fresh weight of *S. miltiorrhiza* shoots and roots under different treatments. The mycorrhizal growth response (MGR) of plants along a P (**b**) or N (**d**) fertilization gradient consisting of four input levels. The means ± standard errors are shown (n = 6). Different lowercase letters indicate significant differences between different treatments according to two-way ANOVA (**a,c**) or one-way ANOVA (**b,d**) followed by Tukey’s test for multiple comparisons (*P* < 0.05).

Applying P fertilizer did not improve the fresh weight of non-mycorrhizal *S. miltiorrhiza* (Fig. 2a). Under the P0, P25, and P50 treatments, mycorrhizal inoculation significantly increased the biomass of shoots, whereas the P100 treatment did not (Fig. 2a). For roots, the growth promoting effect can only be achieved without adding P fertilizer (P0) (Fig. 2a). The P fertilizer addition significantly inhibited the growth of mycorrhizal *S. miltiorrhiza* (Fig. S1a).

The MGR was correlated to the level of P addition, and decreased as P level increased (Fig. 2b). The MGR under P0 treatment was the highest among all P levels, and the MGR under P100 treatment was the lowest. The MGR of shoot biomass decreased in a consistent manner from 379% (P0) to 55% (P100) whereas the MGR of root biomass decreased from 272% (P0) to 12% (P100). The P0 treatment MGR of the whole plant was 15 times higher than that of the P100 treatment (Fig. 2b).

### Low level application of N fertilizer increased the mycorrhizal benefits to plant growth

Under NM treatments, the fresh weight of the shoots or roots did not increase with the N addition dose (Fig. 2c). Regardless of the N addition dose, compared with non-mycorrhizal plants, AMF colonization significantly increased the biomass of *S. miltiorrhiza* shoots and roots (Fig. 2c, S1b). Under mycorrhizal treatments, the fresh weight of shoots increased gradually with increasing N application. However, the fresh weight of roots under N25 was the highest, and a subsequent increase in nitrogen addition decreased the root biomass. Application of a low level of N (N25) resulted in increased biomass of the shoots by 29-fold and that of the roots by 9.4-fold compared with that in non-mycorrhizal plants (Fig. 2c).

Differences in mycorrhizal efficiency were observed at different N levels. Under the N25 treatment, the MGR of both the shoots and roots was the highest, whereas that of the N0 treatment was the lowest (Fig. 2d). Application of a low level of nitrogen fertilizer (N25) is thus conducive to the development of mycorrhizal benefits to plant growth.

### Promotion of bioactive compounds accumulation by AMF was severly inhibited by P fertilizer

**Figure 3:**
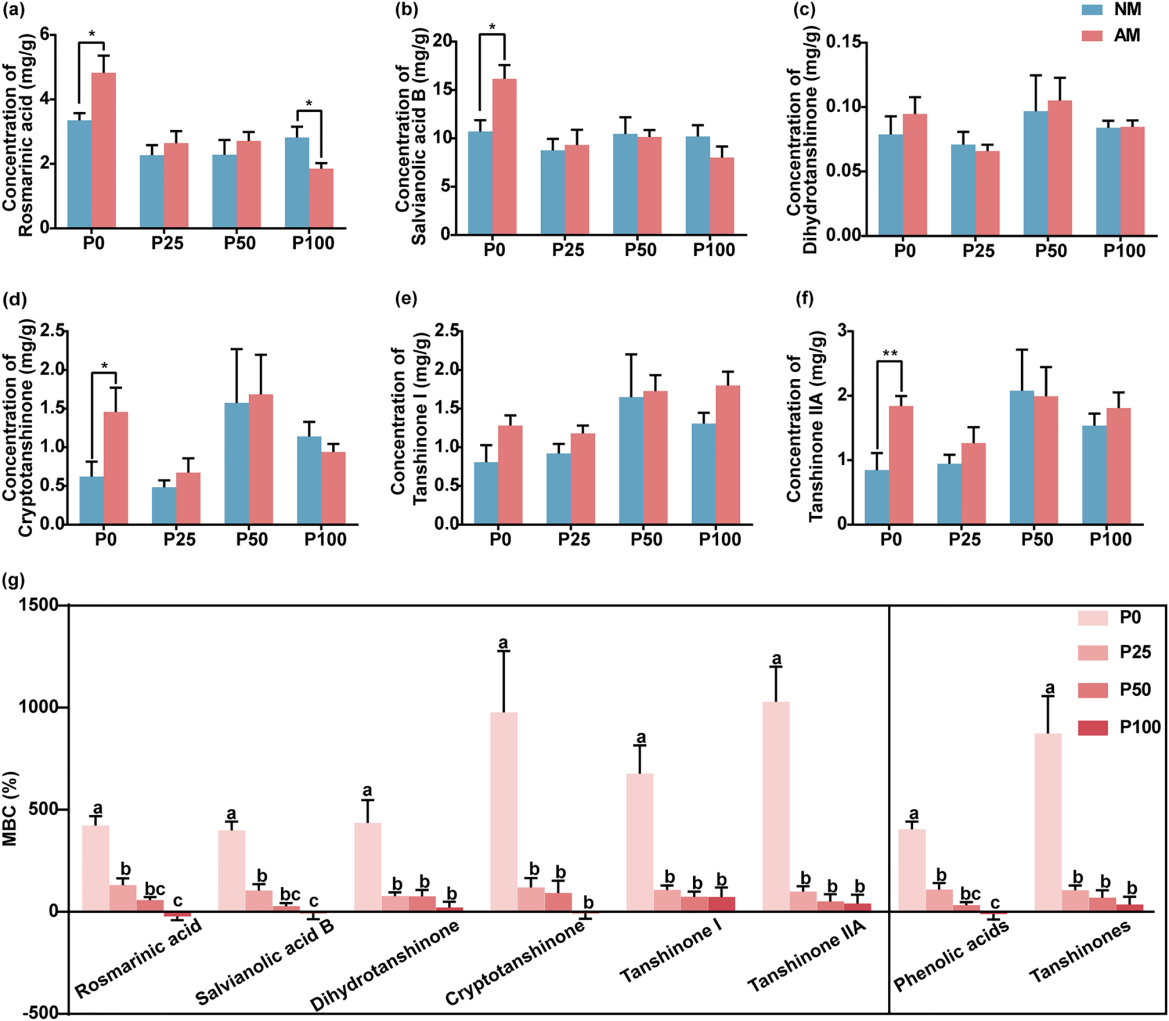
Mycorrhizal response of bioactive compounds (MBC) under different levels of P (0, 25, 50, and 100 mg P fertilizer added per kilogram of soil; P0, P25, P50, P100) fertilizer. (**a–f**) The concentrations of bioactive compounds, rosmarinic acid (**a**), salvianolic acid (**b**), dihydrotanshinone (**c**), cryptotanshinone (**d**), tanshinone I (**e**), and tanshinone IIA (**f**) in the roots of *S. miltiorrhiza* under different treatments. The means ± standard errors are shown (n = 6). Asterisks indicate significant differences between arbuscular mycorrhizal plants and non-mycorrhizal plants according to the t-test (*0.01 ≤ *P* ≤ 0.05, **0.001 ≤ *P* ≤ 0.01, ***P ≤ 0.001). (**g**) Mycorrhizal response of bioactive compounds (MBC) under different levels of P fertilizer. The means ± standard errors are shown (n = 6). Different lowercase letters indicate significant differences between different treatments according to one-way ANOVA followed by Tukey’s test for multiple comparisons (*P* < 0.05).

The bioactive compounds in the roots of *S. miltiorrhiza* include phenolic acids (rosmarinic acid, salvianolic acid B) and tanshinones (dihydrotanshinone, cryptotanshinone, tanshinone I, tanshinone IIA). P fertilizer affected the MBC in the roots of *S. miltiorrhiza*. Under P0 treatment, compared with non-mycorrhizal plants, AMF colonization significantly increased the concentrations of rosmarinic acid by 43.9% (Fig. 3a), salvianolic acid B by 50.9% (Fig. 3b), cryptotanshinone by 134.0% (Fig. 3d), tanshinone IIA by 117.4% (Fig. 3f). Under the P100 treatment, AMF colonization significantly decreased the concentrations of rosmarinic acid by 34.3%. Under the P25, P50, and P100 treatments, there was no significant difference in the concentrations of salvianolic acid B (Fig. 3b), dihydrotanshinone (Fig. 3c), cryptotanshinone (Fig. 3d), tanshinone I (Fig. 3e), and tanshinone IIA (Fig. 3f) between mycorrhizal *S. miltiorrhiza* and non-mycorrhizal *S. miltiorrhiza*.

However, from the perspective of accumulation, mycorrhizal colonization increased the accumulation of almost all bioactive compounds (Fig. 3g). The MBC represents the mycorrhizal response of bioactive compounds, wherein positive values indicate that mycorrhizal colonization could increase the accumulation of bioactive compounds, whereas negative values indicate that mycorrhizal colonization could decrease the accumulation of bioactive compounds. Under the P0, P25, and P50 treatments, mycorrhizal colonization increased the accumulation of all bioactive compounds (Fig. 3g). Under the P100 treatment, mycorrhizal colonization increased the accumulation of dihydrotanshinone, tanshinone I, and tanshinone IIA, but decreased the accumulation of rosmarinic acid, salvianolic acid B, and cryptotanshinone (Fig. 3g). Under the P0 treatment, the MBC of all six bioactive compounds was the highest. The MBC decreased with the level of P addition (Fig. 3g). This indicates that the absence of phosphorus fertilization increased the MBC and that excessive phosphorus addition decreased the MBC.

### Application of N fertilizer increased the MBC of phenolic acids and decreased the MBC of tanshinones

**Figure 4:**
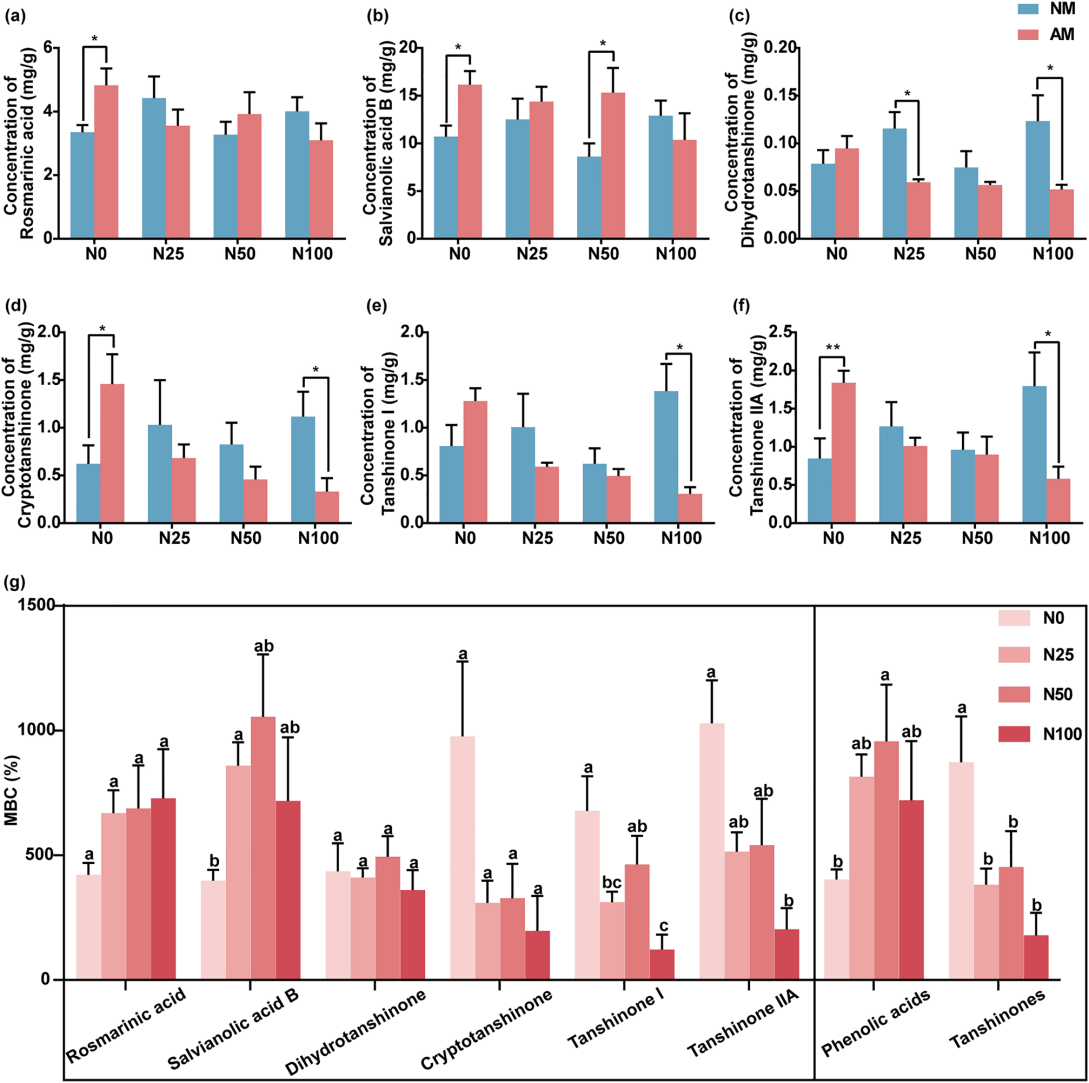
Mycorrhizal response of bioactive compounds (MBC) under different levels of N (0, 25, 50, and 100 mg N fertilizer added per kilogram of soil; N0, N25, N50, N100) fertilizer. (**a–f**) The concentrations of bioactive compounds, rosmarinic acid (**a**), salvianolic acid (**b**), dihydrotanshinone (**c**), cryptotanshinone (**d**), tanshinone I (**e**), tanshinone IIA (**f**) in the roots of *S. miltiorrhiza* under different treaments. The means ± standard errors are shown (n = 6). Asterisks indicate significant differences between arbuscular mycorrhizal plants and non-mycorrhizal plants according to the t-test (*0.01 ≤ *P* ≤ 0.05, **0.001 ≤ *P* ≤ 0.01, ***P ≤ 0.001). (**g**) Mycorrhizal response of bioactive compounds (MBC) under different levels of N. The means ± standard errors are shown (n = 6). Different lowercase letters indicate significant differences between different treatments according to one-way ANOVA followed by Tukey’s test for multiple comparisons (*P* < 0.05).

Without N fertilizer application (the N0 treatment), the concentrations of rosmarinic acid (Fig. 4a), salvianolic acid B (Fig. 4b), cryptotanshinone (Fig. 4d), and tanshinone IIA (Fig. 4f) in mycorrhizal plants was 1.44, 1.51, 2.34, and 2.17 times that in non-mycorrhizal plants. When a high level of N fertilizer was applied (the N100 treatment), mycorrhizal colonization significantly decreased the concentrations of liposoluble constituents such as dihydrotanshinone by 58% (Fig. 4c), cryptotanshinone by 70.0% (Fig. 4d), tanshinone I by 77.7% (Fig. 4e), and tanshinone IIA by 67.55% (Fig. 4f). Overall, AMF increased the concentrations of bioactive compounds in *S. miltiorrhiza* without N fertilizer, but the contrasting result was observed when a high dose of N fertilizer was applied.

Regardless of the N addition dose, mycorrhizal colonization significantly increased the accumulation of bioactive compounds, and the MBC showed all positive values (Fig. 4g). For phenolic acids, a medium dose of N fertilizer application increased the MBC. In contrast, application of N fertilizer decreased the MBC of tanshinones (Fig. 4g).

## Discussion

Numerous researches have proved that AMF have positive effect on growth criteria and phosphorus nutrition, and this effect decreased at higher phosphorus application rates^22^. In this study, the RLC and MGR decreased gradually with the increase in P application (Figs. 1a, 2b), and the MGR of P0 treatment was 15 times higher than that of P100 treatment (Fig. 2b). These findings are consistent with previous reports that P supply represses AMF colonization and mycorrhizal efficiency^23–25^. Shi et al.^26^ mapped a network between transcription factors and mycorrhizal symbiosis-related genes, which is governed by the conserved P-sensing pathway, centered on phosphate starvation response (PHR) transcription factors. They observed the PHR promotes mycorrhizal symbiosis under low P condition, while phosphate sensors, SPX proteins, negatively regulate mycorrhizal symbiosis under high P condition.

AMF colonization is influenced by the available nitrogen contents in soil^27,28^. We observed N application fertilizer significantly increased the RLC, consistent with Wu’s study that N fertilizer increased AMF colonization regardless of the N addition dose^18^. Püschel et al. reported that application of low and medium N levels increased the mycorrhizal efficiency^29^. Similarly, in this study, application of a low level of N (N25) resulted in increased biomass of the shoots by 29-fold and that of the roots by 9.4-fold compared with that in non-mycorrhizal plants (Fig. 2c). A limited supply of N in soil could create competition for N, therefore potentially reducing the net mycorrhizal benefits^19^. N application eliminated the competition between AMF and plants, and significantly improved mycorrhizal colonization and mycorrhizal efficiency.

Secondary metabolites play a critical role in plant defense against biotic and abiotic stresses^30^. The composition and content of secondary metabolites are affected by biological, non-biological, and agronomic management factors^31^. Symbioses between plants and AMFs affect the composition and contents of secondary metabolites e.g., polyphenols, flavonoids, carotenoids, and phytoestrogens, either in crops or medicinal plants^32–34^. Wu et al^35^ showed that AMF inoculation with *S. miltiorrhiza* promoted roots growth and increased secondary metabolites production (especially phenolic acids). In this study, the MBC of all six bioactive compounds was the highest under the P0 treatment. The MBC of phenolic acids decreased from 403.69 to –10.49%, and that of tanshinones decreased from 873.48 to 36.06% (Fig. 3g). The P fertilizer supply severely affected the contribution of mycorrhizal colonization to the phenolic acids and tanshinones of *S. miltiorrhiza*. The reprogramming of plant secondary metabolism due to AMF symbiosis enables the plant to better tolerate abiotic and biotic stresses^36^. Nevertheless, high P condition would inhibit the resistance of plants induced by AMF.

The MBC of phenolic acids and tanshinones had different responses to N fertilizer supply. Our results showed that when a medium dose of N fertilizer was applied (N50), the MBC of phenolic acids was 2.37 times that observed in the N0 treatment; however, the MBC of tanshinones decreased to 50% of that with the N0 treatment (Fig. 4g). The MBC decrease of tanshinones is consistent with the carbon-nutrient balance hypothesis (CNBH). The CNBH presumes that increasing availability of nutrients reduces the concentration of carbon-based secondary metabolites^37^. The MBC increase of phenolic acids in contrast to that observed for CNBH, because the biosynthesis of salvianolic acid B and rosmarinic acid occurs via the shikimate/phenylpropanoid pathway. Phenylalanine and tyrosine are precursors of salvianolic acid B and rosmarinic acid^38^. N is essential for amino acid synthesis. N application could promote the synthesis of phenylalanine and tyrosine, leading to the accumulation of salvianolic acid B and rosmarinic acid in mycorrhizal *S. miltiorrhiza*.

We observed the existence of arbuscular mycorrhiza changed the nutritional demand pattern of *S. miltiorrhiza*. P is the limiting nutrient of non-mycorrhizal plants, while N is the limiting nutrient of mycorrhizal plants. We found significant decrease in the RLC, MGR, and MBC by P fertilizer application, while appropriate N fertilizer application increased the RLC, MGR, and the accumulation of phenolic acids. Nouri et al.^39^ showed the inhibition of P is influenced by other nutritional pathways in the interaction between *Petunia hybrida* and *Rhizophagus irregularis*, and N and P are the major nutritional determinants of the interaction. Interestingly, the symbiosis-promoting effect of nitrogen starvation dominantly overruled the suppressive effect of high phosphorus nutrition onto arbuscular mycorrhiza, suggesting that plants promote the symbiosis as long as they are limited by one of the two major nutrients. Hence, how does N and P fertilizer interactions co-regulate mycorrhizal symbiosis worth further study.

## Conclusion

*S. miltiorrhiza* cultivation requires both AMF inoculation and fertilization to achieve optimal growth and yields; further, AMF application can reduce the use of chemical fertilizers, offering a more sustainable farming system that is environment-friendly. The existence of arbuscular mycorrhiza changed the nutritional demand pattern of *S. miltiorrhiza*. P is the limiting nutrient for non-mycorrhizal plants, while N is the limiting nutrient for mycorrhizal plants. P and N fertilizers affected AMF colonization and mycorrhizal efficiency in *S. miltiorrhiza*. P fertilizer was not conducive to mycorrhizal benefits as the MGR and MBC gradually decreased with increasing P addition. Application of low-dose N fertilizer (N25) significantly increased the MGR, whereas medium-dose N fertilizer (N50) significantly increased the MBC of phenolic acids, but decreased the MBC of tanshinones. In order to obtain the best combination of P and N fertilizers for mycorrhizal cultivation of *S. miltiorrhiza*, the effects of interaction between P and N fertilizers on the mycorrhizal *S. miltiorrhiza* should be studied in the future.

## Methods

### Plant materials, soil, and fungal inoculants

The seeds of *S. miltiorrhiza* used in this study were collected from the Laiwu Danshen cultivation base in Shandong Province, China (36°20′N, 117°41′E), and complied with relevant institutional, national, and international guidelines and legislation. We have obtained the permission to collect seeds.

Soil used for the pot experiment was collected from the top 0‒20 cm of soil at the Laiwu Danshen cultivation base. The soil was sieved to < 4 mm and was then sterilized at 121 °C for 60 min, for 7 consecutive days before initiating the experiment. Soil from this site was classified as weathered rock (50% rock) with the following values: pH 8.44, organic material 4.94 g∙kg^−1^, total nitrogen 240 mg∙kg^−1^, total phosphorus 380 mg∙kg^−1^, total potassium 18,400 mg∙kg^−1^, available nitrogen 35.1 mg∙kg^−1^, available phosphorus 2.3 mg∙kg^−1^, and available potassium 28 mg∙kg^−1^. The contents of total N, P, K in the samples were analyzed, i.e., Kjeldahl method for total N, vanadium molybdate blue colorimetric method for total P, flame photometry for total K. Moreover, hydrolyzable N, available P, available K in soil was quantified using alkaline hydrolysis-diffusion method, ammonium acetate extraction-flame photometry, sodium bicarbonate extraction-vanadium molybdate blue colorimetric method, respectively^40^.

Mycorrhizal inocula were obtained from the sand cultures of *Glomus versiforme*, which were originally provided by Professor Honggang Wang (Beijing Chinese Academy of Agricultural Sciences, China). White clover (*Trifolium repens*) was used as the host. The inocula contained sand, spores, hyphae, and colonized roots of white clover with an average concentration of 20 spores∙g^−1^.

## Experimental design

The pot experiment with cultivated *S. miltiorrhiza* was conducted in a greenhouse in Beijing City, China (116°43′E, 39°59′N). The seeds were collected at the cultivation base mentioned before. To avoid any competing organisms, seeds were first surface-sterilized before sowing as follows: submerging in 75% ethanol for 2 min, then transfer to a 10% H_2_O_2_ solution for 10 min, washing with tap water for 3 min, and finally soaking in sterile water at room temperature for 12 h. The sterilized seeds were then sown in pots containing moist sterilized vermiculite. Two seedlings were transplanted into each experimental pot after 30 days from the time of sowing. Experiments were carried out in an environmentally controlled growth room (30 °C, 14 L:10D photoperiod), with a photon flux density of 350 photon µmol·m^−2^·s^−1^ (photosynthetic active radiation) at the plant-canopy level.

The amount of P and N fertilizer used for the field cultivation of *S. miltiorrhiza* is generally 50 mg calcium superphosphate and urea per kilogram of soil (150 kg∙ha^−1^); therefore, three fertilization treatments including high, medium, and low doses were designed. Four concentrations of P fertilizer (natural baseline amount and further addition of 25, 50, and 100 mg calcium superphosphate per kilogram of soil) were applied with or without AMF inoculation as the P0, P25, P50, and P100 treatments, respectively. Four concentrations of N fertilizer (natural baseline amount and further addition of 25, 50, and 100 mg urea per kilogram of soil) were applied with or without AMF inoculation as the N0, N25, N50, and N100 treatments, respectively. Thus, there were 16 treatments in our experiment. As each treatment had 6 replicates, there were 96 pots in the experiment. Fully mixed the calcium superphosphate or urea with the soil and putted them into pots. The AMF treatments (AM) were inoculated with 10 g of inoculum layered below the seeds in each pot, and the non-mycorrhizal treatments (NM) received 10 g of autoclaved inoculum. Plants were watered with distilled water when necessary and incubated at a temperature of 22‒35 °C. The pots were randomly arranged in the greenhouse. The plants were harvested at 4 months after transplantation, and the fresh weights of the shoots and roots were recorded. The parameters of the MGR and MBC were calculated as (AM-NM_mean_)/NM_mean_∙100%^24^, where AM denotes the value (fresh weight of shoots or roots, accumulation of bioactive compounds in roots) of inoculated plants in the AM treaments and NM_mean_ is the mean value of non-inoculated plants in the corresponding NM treatments.

### Estimation of AMF colonization

Fresh fibrous root samples were used to visualize AMF colonization. To determine AMF colonization, the fibrous roots of *S. miltiorrhiza* were cut into small pieces (approximately 1 cm long). The roots were then stained with Trypan Blue following the procedure proposed by Phillips and Hayman^41^. AMF colonization was determined using the method described by Giovannetti and Mosse^42^.

### Assessment of plant growth

After harvesting, the above and below-ground portions were separated, and the fresh weights of both the aerial portion and roots were recorded.

### Quantification of bioactive compounds in roots

The concentrations of rosmarinic acid (Fig. 1a), salvianolic acid B (Fig. 1b), dihydrotanshinone (Fig. 1c), cryptotanshinone (Fig. 1d), tanshinone I (Fig. 1e), and tanshinone IIA (Fig. 1f) in the roots were determined using the methods described by Chen et al.^15^. Roots harvested from experimental plants were dried at 40 °C for 48 h. The dried roots were ground in a standard grinder and then passed through a 40-mesh sieve. Then, 10 mL of methanol: water solution (80:20) was used to extract the sieved root (0.1 g) in an ultrasonic bath for 30 min at room temperature. After extraction, the solution was filtered through a 0.45 µm filter, and the filtrate was collected. A 10 µL aliquot of the filtrate was injected, and separated using HPLC with a C18 Symmetry^®^ column (4.0 mm × 250 mm, 3 µm; Waters Corp., Milford, MA, United States). The mobile phase A comprised a water-phosphoric acid solution (A; 100:0.10, v/v), and the mobile phase B was acetonitrile. The gradient elution program was as follows: (0–15) min, 75% B; (15–16) min, 25% → 40% B; (16–18) min, 40% → 50% B; (18.0–55.0) min, 50% → 70% B; (55.0–65.0) min, 75% → 25% B. Eluted compounds were detected spectrophotometrically at 280 nm using a 996 PDA photodiode array detector (Waters Corp., Milford, MA, United States). The column temperature was set at 25 °C; the flow rate was 1.0 mL∙min^−1^.

### Statistical analysis

All data were analyzed using IBM SPSS Statistics 24. Results were presented as the mean ± SD of six replicates. The data of the RLC, MGR, and MBC were analysed by one-way ANOVA followed by Tukey’s test for multiple comparisons. The data of the fresh weight of shoots and roots were analysed by two-way ANOVA followed by Tukey’s test for multiple comparisons.The data of the contents of bioactive compounds were subjected to the t-test. Differences were reported as significant when *P* < 0.05.

## Supplementary Information

Supplementary Figure S1.
