# Supplementary material for: Arbuscular mycorrhiza alters the nutritional requirements in Salvia miltiorrhiza and low nitrogen enhances the mycorrhizal efficiency
Source: Sci Rep. 2022 Nov 16;12:19633. doi: 10.1038/s41598-022-17121-2 (PMC9668911; doi:10.1038/s41598-022-17121-2)
Supplement: Supplementary file 1 — Supplementary Figure S1. [file 41598_2022_17121_MOESM1_ESM.docx]

**Supplementary figure**


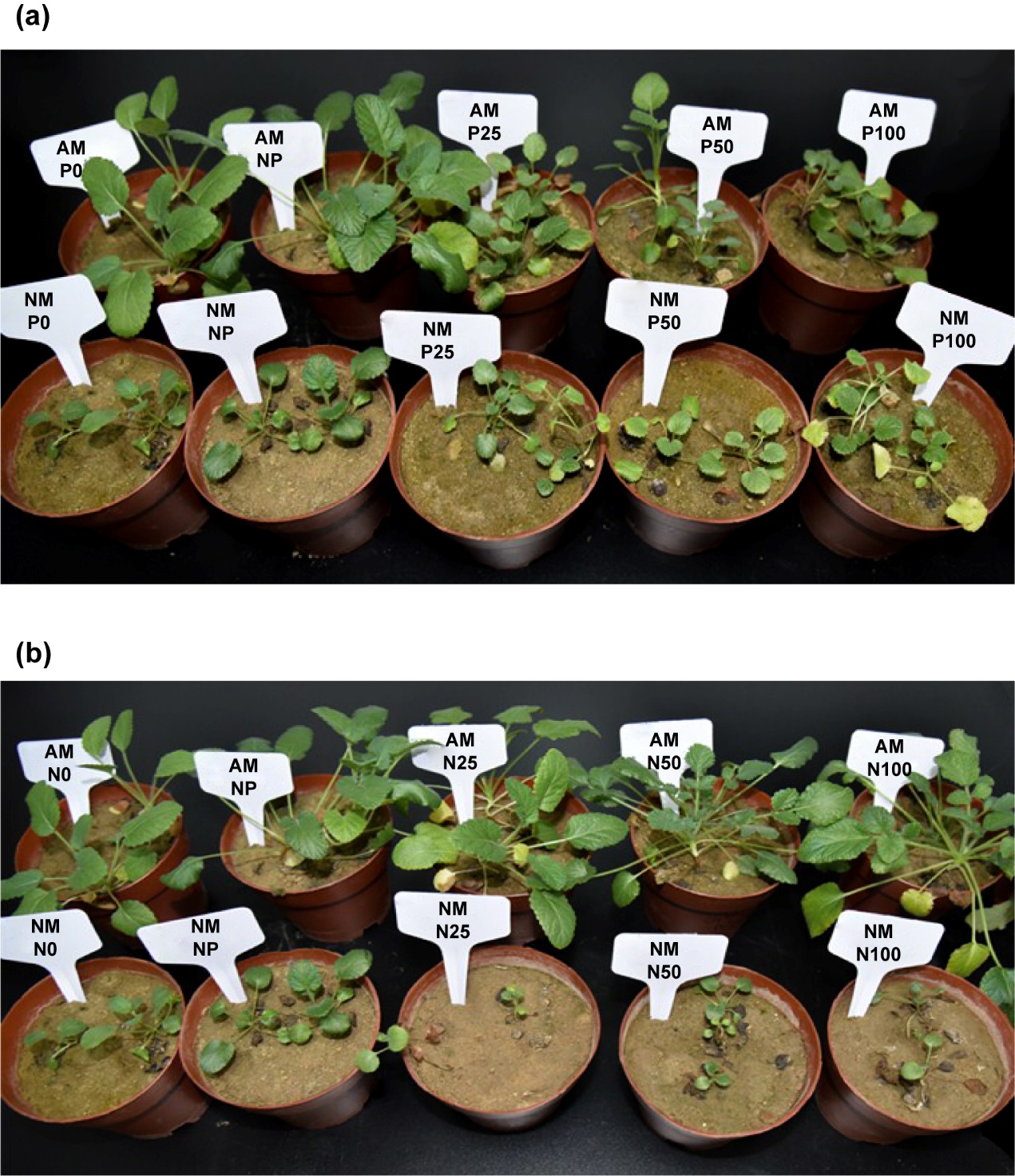


**Figure S1:** (a) Mycorrhizal (AM) and non-mycorrhizal (NM) *S. miltiorrhiza* applied with different amount of P fertilizer. (b) Mycorrhizal *S. miltiorrhiza* (AM) and non-mycorrhizal (NM) *S. miltiorrhiza* applied with different amount of N fertilizer. Photos were taken 120 d after AMF inoculation.
